# Invariant nature of substituted element in metal-hexacyanoferrate

**DOI:** 10.1038/s41598-017-13719-z

**Published:** 2017-10-16

**Authors:** Hideharu Niwa, Wataru Kobayashi, Takayuki Shibata, Hiroaki Nitani, Yutaka Moritomo

**Affiliations:** 1 Faculty of Pure and Applied Science, University of Tsukuba, Tsukuba, 305-8571 Japan; 2 Tsukuba Research Center for Interdisciplinary Materials Sciences (TIMS), University of Tsukuba, Tsukuba, 305-8571 Japan; 3 National Institute of Technology, Gunma College, Maebashi, Gunma, 371-8530 Japan; 4 Institute of Materials Science, High Energy Accelerator Research Organization (KEK), Tsukuba, 305-0801 Japan

## Abstract

The chemical substitution of a transition metal (*M*) is an effective method to improve the functionality of materials. In order to design the highly functional materials, we first have to know the local structure and electronic state around the substituted element. Here, we systematically investigated the local structure and electronic state of the host (*M*_h_) and guest (*M*_g_) transition metals in metal-hexacyanoferrate (*M*-HCF), Na_*x*_(*M*_h_, *M*_g_)[Fe(CN)_6_]_*y*_ (1.40 < *x* < 1.60 and 0.85 < *y* < 0.90), by means of extended X-ray absorption fine structure (EXAFS) and X-ray absorption near-edge structure (XANES) analyses. The EXAFS and XANES analyses revealed that the local structure and electronic state around *M*_g_ are essentially the same as those in the pure compound, *i.e*, *M*_g_-HCF. Such an invariant nature of *M*_g_ in *M*-HCF is in sharp contrast with that in layered oxide, in which the *M*_g_ valence changes so that local *M*_g_-O distance (*d*_*M*-O_^g^) approaches the *M*_h_-O distance (*d*_*M*-O_^h^).

## Introduction

The chemical substitution of transition metal (*M*) is an effective method to improve the functionality of materials, such as electrochemical performance in sodium-ion secondary batteries (SIBs) and critical temperature of magnetic phase transition. Actually, the rate and cycle properties of manganese hxacyanoferrate (Mn-HCF) in SIBs are significantly improved by partial substitution of Fe, Co, and Ni for Mn^1–3^. In addition, the critical temperature for the spin-crossover transition of [Fe_1-*x*_Zn_*x*_ (ptz)_6_](BF_4_)_2_ decreases with the Zn concentration (*x*)^4^. To thoroughly comprehend the partial substitution effect on material properties, we first have to know the local structure and electronic state around substituted guest element (*M*_g_). The extended X-ray absorption fine structure (EXAFS) and X-ray absorption near-edge structure (XANES) analyses around the K-edges are powerful tools to investigate the local structure and electronic state around *M*_g_ and *M*_h_ in the mixed compounds. Here, let us consider the network material, such as coordination polymer and transition metal oxides, in which ligand-bridged *M* chains form one-, two-, or three-dimensional network. For example, in metal hexacyanoferrate (*M*-HCF), Na_*x*_ *M*[Fe(CN)_6_]_*y*_, which is the most prototypical coordination polymer, cyano-bridged -*M*-NC-Fe-CN-*M*-NC- Fe-CN-*M*-NC- chains form three-dimensional (3D) jungle-gym type network. In such a network material, the substitution of *M*_g_ for the host element (*M*_h_), -*M*_h_ -*L*-*M*_h_-*L-M*_g_-*L*-*M*_h_-*L*-*M*_h_- (*L* represents ligand), causes local distortion, *i.e*., elongation or compression of the *M*_g_-L distance (*d*_*M*-L_^g^) reflecting the difference in the ionic radii (*r*) between *M*_g_ and *M*_h_. Such a lattice distortion increases the Gibbs free energy of the system. To minimize the Gibbs free energy, the local distortion should relax via variation in the electronic state of *M*_g_. Actually, in layered oxide system^1^, the *M*_g_ valence changes so that local *M*_g_-O distance (*d*_*M*-O_^g^) approaches the *M*_h_-O distance (*d*_*M*-O_^h^).

Among the network material, the *M*-HCF is the oldest compound that the human being has synthesized. The *M*-HCF consists of a 3D jungle-gym type -*M*-NC-Fe-CN-*M*-NC- Fe-CN-*M*-NC- network and Na^+^ and H_2_O, which are accommodated in the network nanopores. We note that the *M*-HCFs have considerable Fe(CN)_6_ vacancies, where H_2_O molecules coordinated to *M* instead of CN. Most of the *M*-HCFs show the face-centered cubic (*Fm*3(−)*m*; *Z* = 4)^5^ or trigonal (*R*3(−)*m*; *Z* = 3)^6,7^ structures. The *M*-HCFs are attracting current interest of material scientists, because they are promising materials for the lithium-ion secondary batteries (LIBs)^8–10^, SIBs^2,3,11–20^, electrochromism^21,22^, and thermoelectrics^23^. In SIBs, the rate and cycle properties of Mn-HCF are significantly improved by partial substitution of Fe, Co, and Ni for Mn^2,3^. Thus, *M*-HCF is a suitable system for investigation of the local structure and electronic state around *M*_g_ and *M*_h_.

Here, we systematically investigated the local structure and electronic state around *M*_g_ and *M*_h_ in Na_*x*_(*M*_h_, *M*_g_)[Fe(CN)_6_]_*y*_ (1.40 < *x* < 1.60 and 0.85 < *y* < 0.90) by means of the EXAFS and XANES analyses. The analyses revealed that the local structure and electronic state of *M*_g_ are essentially the same as that in the pure material, *i.e*., *M*_g_-HCF. Such an invariant nature of *M*_g_ in *M*-HCF is in sharp contrast with that in layered oxide, in which the *M*_g_ valence changes so that *d*_*M*-O_^g^ approaches *d*_*M*-O_^h^. We will discuss the origin for the difference in the substitution effect between *M*-HCF and the layered oxide.

## EXAFS analysis

We performed careful EXAFS analysis on three pure (*M*-HCF) and six mixed (*M*_h_ *M*_g_-HCF) compounds. Details of synthesis and characterization are described in the Method section. The x-ray diffraction (XRD) patterns for the nine compounds are shown in Fig. S1. In the EXAFS analyses, we included the contributions from the first- (N) and second- (C) nearest neighbor elements. In order to include the Fe(CN)_6_ vacancy effect, the coordination numbers (*N*_N_) of N are treated as an adjustable parameter with restriction of *N*_N_ + *N*_O_ = 6 and *d*_*M*-O_ = *d*_*M*-N_, where *N*_o_ is the coordination number of O. With use of the EXAFS equation, least-squares fittings are performed for the FT[*χ*(*k*)*k* ^3^]−*R* plots (Fig. S2). Thus obtained interatomic distances are listed in Table 1.

**Table 1:** Interatomic distances, *d*_*M*-N_ and *d*_*M*-C_, in pure (*M*-HCF: Na_*x*_ *M*[Fe(CN)_6_]_*y*_) and mixed (*M*_h_ *M*_g_-HCF: Na_*x*_(M_h_, M_g_)[Fe(CN)_6_]_*y*_) compounds.

Compound	*d*_*M*-N_^pure^(Å)	*d*_*M*-N_^h^(Å)	*d*_*M*-N_^g^(Å)	*d*_*M*-C_^pure^(Å)	*d*_*M*-C_^h^(Å)	*d*_*M*-*C*_^g^(Å)
Mn-HCF	2.217(5)			3.382(6)		
MnCo-HCF		2.210(7)	2.105(28)		3.377(7)	3.284(24)
MnNi-HCF		2.209(5)	2.100(8)		3.378(6)	3.265(11)
Co-HCF	2.100(8)			3.273(9)		
CoMn-HCF		2.103(8)	2.202(6)		3.276(8)	3.368(8)
CoNi-HCF		2.097(9)	2.088(8)		3.272(10)	3.253(11)
Ni-HCF	2.085(11)			3.237(11)		
NiMn-HCF		2.089(9)	2.197(6)		3.243(10)	3.359(8)
NiCo-HCF		2.085(11)	2.081(30)		3.238(11)	3.251(43)

Note that *M*-HCF and *M*_h_ *M*_g_-HCF have Fe(CN)_6_ vacancies (0.85 < *y* < 0.90). The actual chemical compositions are shown in Table 2. Superscript, pure, in *d*_*M*-N_ and *d*_*M*-C_ means pure compound. Superscripts, h an*d* g, in d_*M*−N_ and *d*_*M*−C_ mean host and guest elements, respectively.

## Interatomic distance between *M* and N

**Figure 1:**
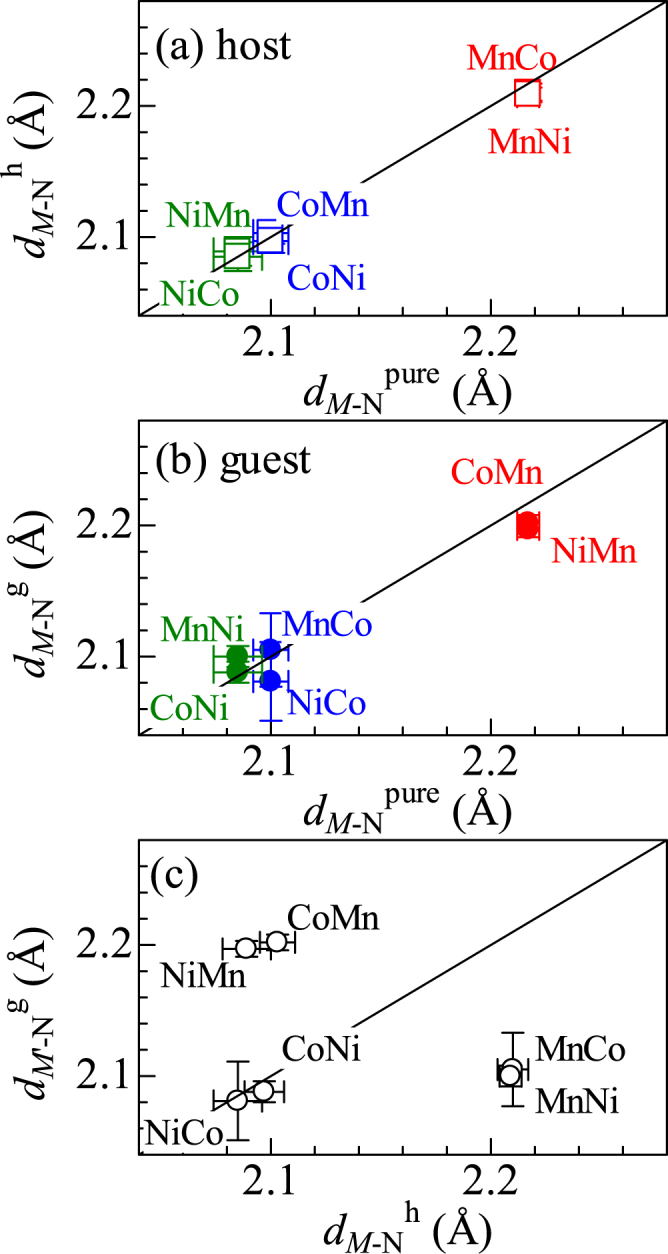
(**a**) Correlation diagram of the *M*−N distances (*d*_*M*-N_) between the mixed compounds (*d*_*M*-N_^h^) around *M*_h_ and pure compounds (*d*_*M*−N_^pure^). (**b**) Correlation diagram of *d*_*M*-N_ between the mixed compounds (*d*_*M*-N_^g^) around *M*_g_ and *d*_*M*-N_^pure^. (**c**) Comparison between *d*_*M*-N_^g^ and *d*_*M*-N_^h^ in the mixed compounds. *M*_h_ *M*_g_ represents Na_*x*_(*M*_h_, *M*_g_)[Fe(CN)_6_]_*y*_. Red, blue and green colors represent the interatomic distances around Mn, Co, and Ni, respectively.

Figure 1(a) shows correlation diagram of the *M*-N distances (*d*_*M*-N_) between the mixed compounds (*d*_*M*-N_^h^) around *M*_h_ and pure compounds (*d*_*M*-N_^pure^). We found that *d*_*M*-N_^h^ in the mixed compound is the same as *d*_*M*-N_^pure^ in the pure compound, indicating that partial substitution of *M*_g_ for *M*_h_ has no effect on *d*_*M*-N_^h^. Figure 1(b) shows correlation diagram of *d*_*M*-N_ between the mixed compound (*d*_*M*-N_^g^) around *M*_g_ and *d*_*M*-N_^pure^. Importantly, *d*_*M*-N_^g^ in the mixed compound is the same as *d*_*M*-N_^pure^.

**Figure 2:**
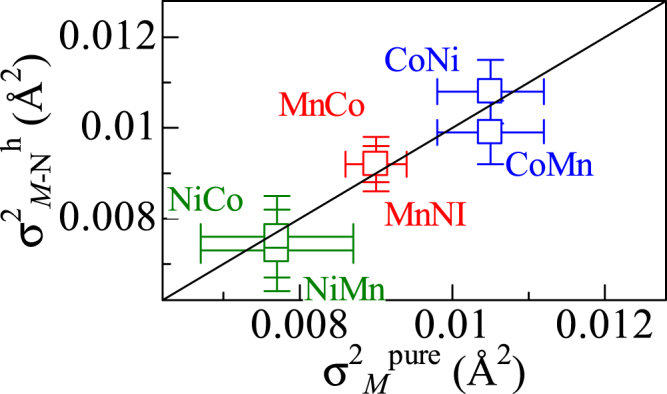
Correlation diagram of the Debye–Waller factor (*σ* ^2^_*M−N*_) between the mixed compounds (*σ* ^2^_*M−N*_^h^) around *M*_h_ and pure compounds (*σ* ^2^_*M−N*_^pure^). *M*_h_ *M*_g_ represents Na_*x*_(*M*_h_, *M*_g_)[Fe(CN)_6_]_*y*_. Red, blue and green colors represent the values around Mn, Co, and Ni, respectively.

As-mentioned above, *d*_*M*-N_^g^ (*d*_*M*-N_^h^) is the same as that in *M*_g_-HCF (*M*_h_-HCF). Such an invariant nature causes significant difference between *d*_*M*-N_^h^ and *d*_*M*-N_^g^. In Fig. 1(c), we compared *d*_*M*-N_^h^ and *d*_*M*-N_^g^ in the mixed compounds. In CoMn-, NiMn-, MnCo-, and MnNi-HCFs, we observed significant difference between *d*_*M*-N_^h^ and *d*_*M*-N_^g^. The difference (|*d*_*M*-N_^h^ − *d*_*M*-N_^g^| = 0.1 Å) corresponds 5% of *d*_*M*-N_. If such a local distortion around *M*_g_ influences the *d*_*M*-N_^h^ value at the nearest-neighbor *M*_h_ site, the partial substitution causes significant distribution of *d*_*M*-N_^h^. Such a distribution of *d*_*M*-N_^h^ should increase the Debye–Waller factor (*σ* ^2^_*M-N*_^h^) around *M*_h_. Figure 2 shows correlation diagram of *σ* ^2^_*M-N*_ between the mixed compounds (*σ* ^2^_*M-N*_^h^) around *M*_h_ and pure compounds (*σ* ^2^_*M-N*_^pure^). *σ* ^2^_*M-N*_^h^ in the mixed compound is the same as *σ* ^2^_*M-N*_^pure^ in the pure compound. This indicates that the partial substitution of *M*_g_ has no effect even at the nearest-neighbor *M*_h_ site.

## Interatomic distance between *M* and C

**Figure 3:**
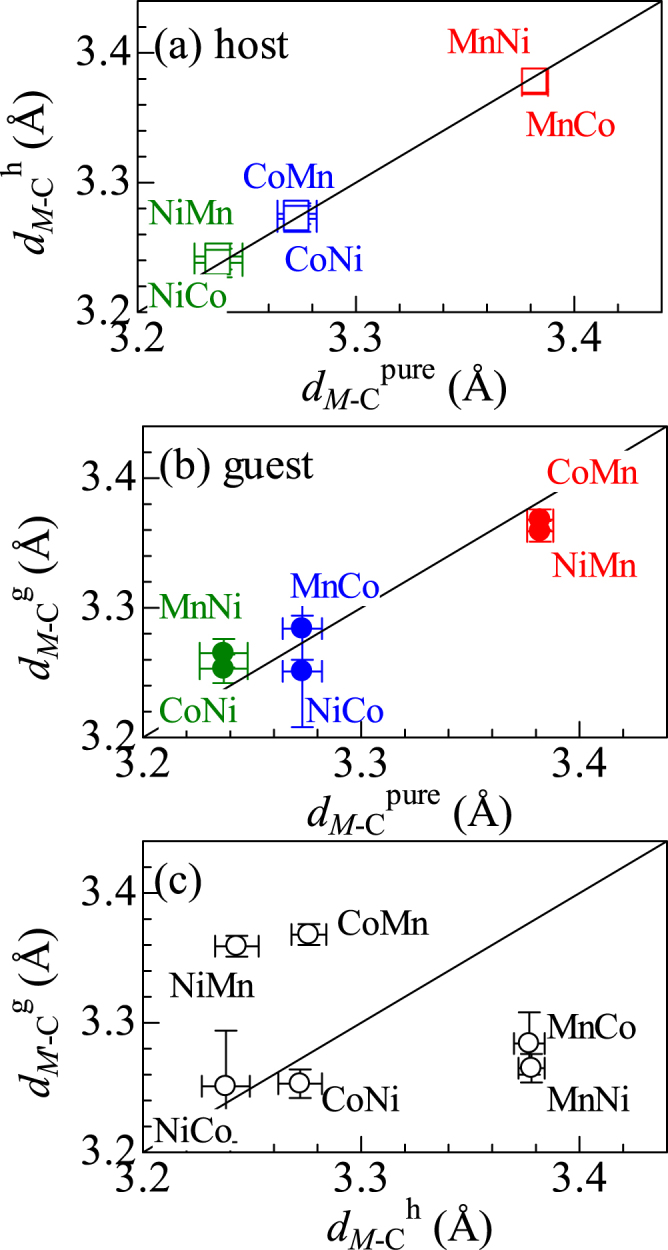
(**a**) Correlation diagram of the *M*−C distances (*d*_*M*-C_) between the mixed compounds (*d*_*M*-C_^h^) around *M*_h_ and pure compounds. (**b**) Correlation diagram of *d*_*M*-C_ between the mixed compounds (*d*_*M*-C_^g^) around *M*_g_ and pure compounds. (**c**) Comparison between *d*_*M*-C_^g^ and *d*_*M*-C_^h^ in the mixed compounds. *M*_h_ *M*_g_ represents Na_*x*_(*M*_h_, *M*_g_)[Fe(CN)_6_]_*y*_. Red, blue and green colors represent the interatomic distances around Mn, Co, and Ni, respectively.

Figure 3(a) shows correlation diagram of the *M* - C distances (*d*_*M*-C_) between the mixed compounds (*d*_*M*-C_^h^) around *M*_h_ and pure compounds (*d*_*M*-C_^pure^). We found that *d*_*M*-C_^h^ in the mixed compound is the same as *d*_*M*-C_^pure^, indicating that partial substitution of *M*_g_ for *M*_h_ has no effect on *d*_*M*-C_^h^. Figure 3(b) is the correlation diagram of *d*_*M*-C_ between the mixed compounds (*d*_*M*-C_^g^) around *M*_g_ and *d*_*M*-C_^pure^. *d*_*M*-C_^g^ in the mixed compound is the same as *d*_*M*-C_^pure^. Here, recall that *d*_*M*-N_^g^ in the mixed compound is the same as *d*_*M*-N_^pure^ [Fig. 1(b)]. These observations indicate that the ligand (CN^-^) environment around *M*_g_ in the mixed compound is the same as that in the pure compound, *i.e*., *M*_g_-HCF. In Fig. 3(c), we compared *d*_*M*-C_^h^ and *d*_*M*-C_^g^ in the mixed compounds. In CoMn-, NiMn-, MnCo-, and MnNi-HCFs, we observed significant difference (|*d*_*M*-C_^h^ − *d*_*M*-C_^g^| = 0.1 Å) between *d*_*M*-C_^h^ and *d*_*M*-C_^g^.

## Electronic state

**Figure 4:**
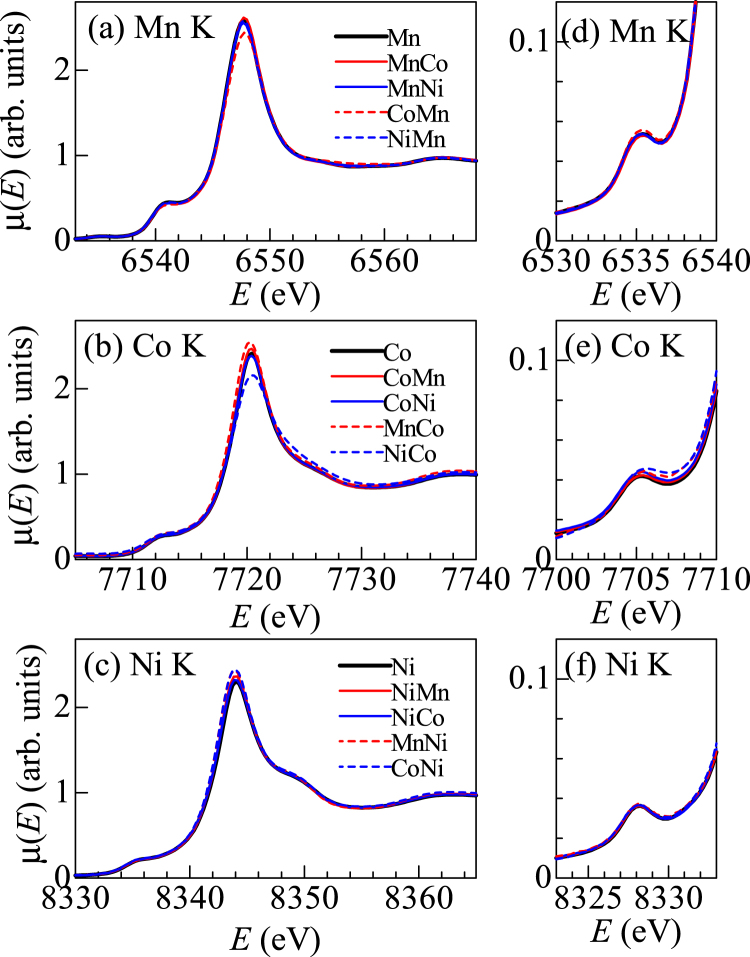
XANES spectra of pure (*M*-HCF) and mixed (*M*_h_ *M*_g_-HCF) compounds around the (**a**) Mn K-edge, (**b**) Co K-edge, and (**c**) Ni K-edge. Thick black curves represent the spectra of pure compounds. Thin solid and broken curves correspond to the spectra around *M*_h_ and *M*_g_ in the mixed compounds, respectively. (**d**)–(**f**) Magnified spectra in the pre-edge region.

Now, let us proceed to the electronic state of *M*_g_ and *M*_h_. Figure 4 shows the XANES spectra of the pure (*M*-HCF) and mixed (*M*_h_ *M*_g_-HCF) compounds around the (a) Mn K-edge, (b) Co K-edge, and (c) Ni K-edge. The thick black curve represents the spectra of pure compounds. The thin solid and broken curves correspond to the spectra around *M*_h_ and *M*_g_ in the mixed compounds, respectively.

In the Mn K-edge spectra [Fig. 4(a)], the main peak is attributable to the Mn1s–4p transition, whose position is a crude measures of the Mn valence. The blue- (red-) shift of the main peak suggests increase (decrease) in the Mn valence. In Mn-HCF (thick black curve), the main peak is observed at 6548.0 eV. We observed no detectable peak-shift in MnCo- and MnNi-HCFs, indicating that the Mn valence is robust against the partial substitution. Importantly, the peak positions of the substituted Mn in CoMn- and NiMn-HCFs are the same as that of Mn-HCF. Figure 4(d) shows magnified Mn K-edge spectra in the pre-edge region, which is dominated by the Mn1s-3d transition. We observed no detectable peak shift in MnCo- and MnNi-HCFs. These observations indicate that electronic states of the substituted Mn in MnCo- and MnNi-HCFs are the same as that in Mn-HCF. This is consistent with the fact that the ligand (CN^−^) environments around substituted Mn in CoMn- and NiMn-HCFs are the same as that in Mn-HCF [Figs 1(b) and 3(b)].

Similarly to the case of the Mn K-edge spectra, we observed no detectable peak-shift in the Co K- and Ni- K-edge spectra. In Fig. 4(b), the peak positions of substituted Co in MnCo- and NiCo-HCFs are the same as that in Co-HCF. In Fig. 4(c), the peak positions of substituted Ni in MnNi- and CoNi-HCFs are the same as that in Ni-HCF. We observed no detectable peak shift in the respective pre-edge region [Fig. 4(e) and (f)]. Thus, the electronic states of the substituted Co and Ni are the same as those in Co- and Ni-HCFs, respectively. This is consistent with the fact that the ligand (CN^−^) environments around substituted Co and Ni are the same as those Co- and Ni-HCFs, respectively [see Fig. 1(b) and Fig. 3(b)].

## Discussion

Now, let us discuss the difference in the local structures between the *M*-HCF and layered oxides. In *M*-HCF, the local structure around *M*_g_ is essentially the same as that in the pure compound. In layered oxide, however, the *M*_g_ valence changes so that *d*_*M*-O_^g^ approaches *d*_*M*-O_^h^. Such a change in *d*_*M*-O_^g^ relaxes the local distortion and lowers the Gibbs free energy of the system. Why is local structure around *M*_g_ invariant in *M*-HCF and is not in layered oxide? We ascribed the invariant nature in *M*- HCF to (1) strong ionicity of [Fe(CN)_6_]^4−^ and (2) the structural flexibility in the jungle-gym type network. First of all, [Fe(CN)_6_]^4−^ is a hard anion with a stable closed electronic configuration. In fact, the profiles of the XANES spectra around Fe K-edge are the same for pure Mn-, Co-, and Ni-HCFs [Fig. 3S(a) and (c)]. Then, it is difficult for *M*_g_ to exchange charge with [Fe(CN)_6_]^4−^. Consistently, the XANES spectra around Fe K-edge hardly change in the mixed compounds [Fig. 3S(b) and (d)]. By means of the EXAFS analyses, we further investigated *d*_Fe-C_ (and *d*_Fe-N_) in the nine compounds (Table S2). We found that *d*_Fe-C_ (and *d*_Fe-N_) is the same for the nine compounds within the experimental errors. By contract, in transition metal oxides, the hybridization between the O 2p and *M* 3d orbitals effectively causes the *M*-dependent charge exchange between O and *M*. Such a hybridization can modify the *M*_g_ valence to minimize the Gibbs free energy. Secondary, the jungle-gym type network of *M*-HCF is flexible against the partial substitution. This is because the network is fairly sparse and has considerable Fe(CN)_6_ vacancies. Actually, the density (~1.9 g/cm_3_) of *M*-HCF is much smaller than that (=5.0 g/cm^3^) of layered oxides. With such a structure, local elongation (compression) of *d*_*M*-N_^g^ in the *M*-NC-Fe-CN-*M*_g_-V-*M*-NC-(V represents the vacancy) chain can be compensated by off-axial rotation of the Fe(CN)_6_ unit (axial displacement of *M*_g_). By contract, in layered oxides, the Na, M, and O ions form respective triangular lattices. The atomic sheets stacks along the *c*-axis in the order of Na, O, *M*, O, and Na. In such a pseudo-close-packed structure, the local distortion induced by *M*_g_ is hard to be compensated.

## Summary

By means of the systematic EXAFS and XANES analyses, we found that the local structure and electronic state of *M*_g_ in *M*-HCF are essentially the same as those in the pure material, *i.e*., *M*_g_-HCF. Such an invariant nature observed in *M*-HCF is in sharp contrast with the layered oxide systems. We ascribed the invariant nature to (1) strong iconicity of [Fe(CN)_6_]_4−_ and (2) the structural flexibility in the jungle-gym type network. Our observation indicates that *M*-HCF is a novel platform of transition metals, in which magnetic, electronic, local structural properties of them are the same as those in the pure compound. With these characteristics, we can easily design and/or calculate the macroscopic magnetic, electric, structural properties of mixed *M*-HCF from the atomic level.

## Method

### Sample preparation and chemical composition

We prepared pure (*M*-HCF; M = Mn, Co, and Ni) and mixed (*M*_h_ *M*_g_-HCF; *M*_h_ and *M*_g_ = Mn, Co, and Ni) compounds by participation method from aqueous solutions in air at 40 °C. In the synthesis of pure compounds, an aqueous solution (40 mM *M*Cl_2_: *M* = Mn, Co, and Ni) was slowly dropped to an aqueous solution (40 mM Na_4_[Fe^II^(CN)_6_] and 4 M NaCl). In the synthesis of mixed compounds, an aqueous solution (32 mM *M*_h_Cl_2_, 8 mM *M*_g_Cl_2_, and 4 M NaCl: *M*_h_ and *M*_g_ = Mn, Co, and Ni) was slowly dropped to an aqueous solution (40 mM Na_4_[Fe^II^(CN)_6_] and 4 M NaCl). In both the cases, the latter solution was stirred at 250 rpm with a magnetic stirrer during the instillation. The dropping rate ( = 100 ml/hour) was controlled with use of a tube pump. After the instillation, the solutions were kept for 12 hours. Then, the precipitates were gathered with a 0.1 μm filter, washed well with distilled water, and dried in air. The colors of the obtained powders were white (Mn-HCF), light yellow (MnNi-HCF), light green (Ni-, MnCo-, and CoMn-HCF), light blue (NiMn-HCF), and dark green (Co-, CoNi-, and NiCo-HCF).

The chemical compositions of the pure compounds were determined so as minimize the trial function:

$${\rm{F}}(y,z)=\sum _{i={\rm{Na}},M,{\rm{Fe}}}{({\text{wt} \% }_{{\rm{obs}}}^{i}-{\text{wt} \% }_{{\rm{cal}}}^{i})}^{2},$$

where wt%_obs_^*i*^ and wt%_cal_^*i*^ are the experimentally-obtained and calculated weight percent of the *i-*th elements, respectively. wt%_obs_^*i*^ for the metal elements were determined by the inductively-coupled plasma (ICP) method. In the calculation, we assume the chemical formula: Na_4*y*−2_ *M*[Fe(CN)_6_]_*y*_ *z*H_2_O. The chemical compositions of the mixed compounds were determined so as minimize the trial function:

$${\rm{F}}(x,y,z)=\sum _{i={\rm{Na}},{M}_{{\rm{g}}},{M}_{{\rm{h}}},{\rm{Fe}}}{({\text{wt} \% }_{{\rm{obs}}}^{i}-{\text{wt} \% }_{{\rm{cal}}}^{i})}^{2}.$$

wt%_obs_^i^ for the metal elements were determined by the ICP method. In the calculation, we assume the chemical formula: Na_4*y*−2_ *M*_g1-*x*_ *M*_h*x*_[Fe(CN)_6_]_*y*_ *z*H_2_O. Thus determined chemical compositions are listed in Table 2.

**Table 2:** Chemical composition and lattice constants of pure (*M*-HCF) and mixed (*M*_h_ *M*_g_-HCF) compounds. *a*_H_ and *c*_H_ are the lattice constants in the hexagonal setting.

Compound	composition	structure	*a* (Å)	*a*_H_ (Å)	*c*_H_ (Å)
Mn-HCF	Na_1.52_Mn[Fe(CN)_6_]_0.88_2.0H_2_O	trigonal	(10.5448)	7.5253(8)	17.9307(20)
MnCo-HCF	Na_1.56_Mn_0.80_Co_0.20_[Fe(CN)_6_]_0.89_1.7H_2_O	trigonal	(10.5165)	7.5237(6)	17.7945(14)
MnNi-HCF	Na_1.64_Mn_0.75_Ni_0.25_[Fe(CN)_6_]_0.91_4.2H_2_O	trigonal	(10.4464)	7.4440(7)	17.8161(16)
Co-HCF	Na_1.40_Co[Fe(CN)_6_]_0.85_3.9H_2_O	trigonal	(10.3620)	7.4240(6)	17.4818(13)
CoMn-HCF	Na_1.40_Co_0.75_Mn_0.25_[Fe(CN)_6_]_0.85_4.5H_2_O	trigonal	(10.4047)	7.4544(5)	17.5545(11)
CoNi-HCF	Na_1.44_Co_0.75_Ni_0.25_[Fe(CN)_6_]_0.86_4.6H_2_O	trigonal	(10.3458)	7.4133(7)	17.4502(16)
Ni-HCF	Na_1.52_Ni[Fe(CN)_6_]_0.88_5.8H_2_O	cubic	10.2887(21)		
NiMn-HCF	Na_1.60_Ni_0.78_Mn_0.22_[Fe(CN)_6_]_0.90_4.7H_2_O	cubic	10.3455(24)		
NiCo-HCF	Na_1.48_Ni_0.82_Co_0.18_[Fe(CN)_6_]_0.87_5.5H_2_O	cubic	10.2982(21)		

The lattice constants in the brackets are approximate values (=2^1/3^/3^1/6^ *a*_H_^2/3^ *c*_H_^1/3^) in the pseudo-cubic setting.

### Crystal structure and lattice constants

**Figure 5:**
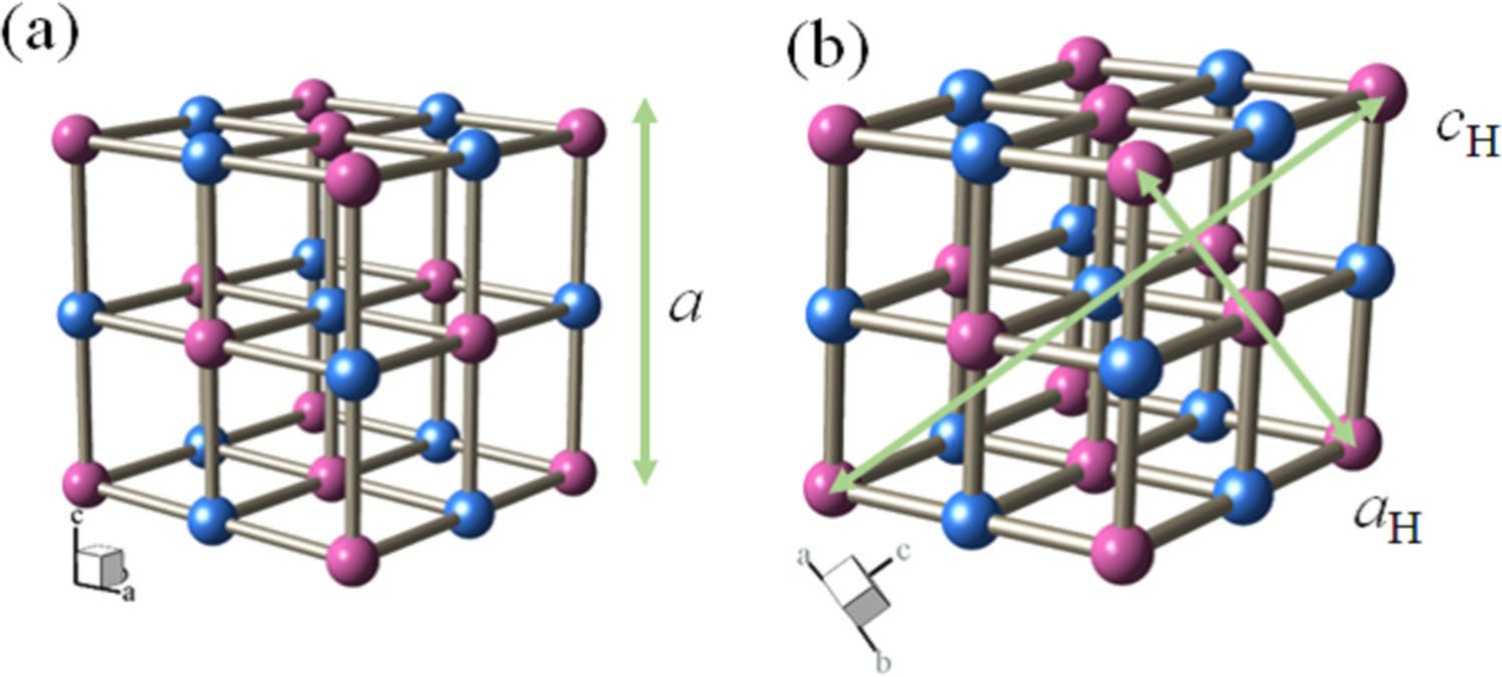
Schematic pictures of (**a**) fcc and (**b**) trigonal (hexagonal setting) structures. Red and blue spheres represents Fe and *M*, respectively. Bars represent CN. Na and H_2_O are omitted for simplicity.

Synchrotron-radiation XRD measurements were performed at the BL8A beamline of the Photon Factory, KEK. The wavelength (=0.689028 Å) of the X-ray was calibrated by the lattice constant of standard CeO_2_ powders. The samples were finely ground and placed in 0.3 mmϕ glass capillaries. The capillaries were sealed and mounted on the Debye-Scherrer camera. The XRD patterns (Fig. S1) were detected with an imaging plate. The exposure time was 5 minutes. The XRD patterns of Ni-, NiCo-, and NiMn-HCFs can be indexed in the face-centered cubic (fcc) structure (*Fm*3(−)*m*; *Z* = 4). The lattice constants, *a*, were refined by the Rietveld method (Rietan-FP^24^). The XRD patterns of Co-, CoMn-, CoNi-, Mn-, MnCo-, and MnNi-HCFs can be indexed in the trigonal structure (*R*3(−)*m*; *Z* = 3).The lattice constants, *a*_H_ and *c*_H_, were refined in the hexagonal setting. The lattice constants, *a*, *a*_H_, and *c*_H_, are listed in Table 2. Figure 5 shows schematic pictures of the (a) fcc and (b) trigonal (hexagonal setting) structures. The two structures are essentially the same expect for the slight elongation along the (111) direction in the trigonal structure.

### X-ray absorption spectroscopy

The X-ray absorption spectroscopy (XAS) measurements were conducted at BL-9C of the Photon Factory, KEK. The powder was finely ground, mixed with BN, and pressed into pellets with 5 mm in diameter. The XAS were recorded in a transmission mode with a Si(111) double-crystal monochromator at 300 K. The wavelengths of the monochromator were calibrated with the absorption edge of Fe, Mn, Co, and Ni foil. In X-ray absorption near edge structure (XANES) analyses, the background subtraction and normalization were performed using the ATHENA program^25^.

### EXAFS analysis

After the background subtraction and normalization of the XAS spectra, oscillatory components *χ* were extracted against the angular wavenumber *k*. *k* is defined by $k=\sqrt{2m(E-{E}_{o})/\hslash }$ where *m*, *E*, and *E*_0_ are the electron mass, energy of the incident X-ray, and absorption edge energy, respectively. Then, Fourier transformation of *χ*(*k*)*k* ^3^ was performed in the *k*-range from 3.0 to 11.0 Å^−1^ These data procedures were performed with use of ATHENA program^25^.

In order to refine the structural parameters, least-squares fittings are performed for the FT[*χ*(*k*)*k* ^3^] − *R* plots with use of ARTEMIS program^25^. In the plane wave and single-scattering approximation, *χ*(*k*) around the K-edge is expressed by the EXAFS equation as:

$${\rm{\chi }}(k)=-{S}_{0}^{2}\sum _{j}\frac{{N}_{j}}{k{R}_{j}^{2}}{F}_{j}(k){e}^{-2{\sigma }_{j}{k}^{2}}\,\sin \,\{2k{R}_{j}+{\phi }_{j}(k)\},$$

where *S*_0_, *N*_j_, *R*_j_, *F*_j_, *σ*_j_^2^, and *φ*_j_ are the passive electron reduction factor, degeneracy of path, path length, effective scattering amplitude, mean square displacement, and effective scattering phase shift of the *j*th atom, respectively. The least-squares fittings were performed in the *R* range from 1 Å to 3.2 Å. *S*_0_^2^ for each absorption edge were estimated from the pure compounds: *S*_0_^2^(Fe) = 0.92, *S*_0_^2^(Mn) = 0.97, *S*_0_^2^(Co) = 1.09, and *S*_0_^2^(Ni) = 1.03. These *S*_0_^2^ values were use in the analyses of the mixed compounds. Practically speaking, *S*_0_^2^ lies within a general range between 0.7 and 1.1^26^. In this sense, the other structural parameters obtained the EXAFS analysis are valid, even though *S*_0_^2^ > 1 is physically meaningless. In the analyses around the Mn, Co, and Ni K-edges, we included the contributions from the first- (N) and second- (C) nearest neighbor elements. Because of the linear _*M*-N_-C-Fe coordination, single (*M*-C-*M*), double (*M*-C-N-*M*), and triple (M-N-C-N-*M*) scattering paths were taken into account in the analyses of second nearest neighbor elements (C). In order to include the Fe(CN)_6_ vacancy effect, *N*_N_ are treated as an adjustable parameter with restriction of *N*_N_ + *N*_O_ = 6 and *d*_*M*-O_ = *d*_*M*-N_. Figure 2S shows prototypical examples of the fitting. The obtained structural parameters are listed in Table S1.In the analyses around the Fe K-edge, we included the contributions from the first- (C) and second- (N) nearest neighbor elements. Because of the linear Fe-C-N-*M* coordination, single (Fe-N-Fe), double (Fe-N-C-Fe), and triple (Fe-C-N-C-Fe) scattering paths were taken into account in the analyses of second nearest neighbor elements (N). We fixed *N*_C_ and *N*_N_ at 6. The obtained structural parameters are listed in Table S2.

## Electronic supplementary material

Supporting information

## References

[1] Akama S (2017). Local structures around the substituted elements in mixed layered oxides. Sci. Rep..

[2] Yang D (2014). Structure optimization of prussian blue analogue cathode materials for advanced sodium ion batteries. Chem. Commum..

[3] Moritomo Y, Urase S, Shibata T (2016). Enhanced battery performance in manganese Hexacyanoferrate in partial substitution. Electrochem. Acta..

[4] Hauzer A (2004). Light-induced spin crossover and the high-spin → low-spin relaxation. Top Curr Chem.

[5] Matsuda T, Kim JE, Ohoyama K, Moritomo Y (2009). Universal thermal response of Prussian blue lattice”. Phys. Rev..

[6] Moritomo Y, Kurihara Y, Matsuda T, Kim JE (2016). Cubic-Rhombohedral Structural Phase Transition in Na_1.32_Mn[Fe(CN)_6_]_0.83_3.6H_2_O. J. Phys. Soc. Jpn..

[7] Moritomo Y, Kurihara Y, Matsuda T, Kim JE (2011). Structural phase diagram of Mn-Fe cyanide against cation concentration. J. Phys. Soc. Jpn..

[8] Imanishi N (1999). Lithium intercalation behavior into iron cyanide complex as positive electrode of lithium secondary battery. J. Power Source.

[9] Imanishi N (1999). Lithium intercalation behavior in iron cyanometallates. J. Power Source.

[10] Matsuda T, Moritomo Y (2011). Thin film electrode of Prussian blue analogue for Li-ion battery”. Appl. Phys. Express.

[11] Lu Y, Wang L, Cheng J, Goodenough JB (2012). Prussian blue: a new framework of electrode materials for sodium batteries. Chem. Commun..

[12] Matsuda T, Takachi M, Moritomo Y (2013). A sodium manganese ferrocyanide thin film for Na-ion batteries. Chem. Commun..

[13] Takachi M, Matsuda T, Moritomo Y (2013). Cobalt hexacyanoferrate as cathode material for Na^+^ secondary battery. Appl. Phys. Express.

[14] Lee HW (2014). Manganese hexacyanomanganate open framework as a high-capacity positive electrode material for sodium-ion batteries. Nature Commun..

[15] Wang L (2015). Rhombohedral prussian white as cathode for rechargeable sodium-ion batteries. J. Am. Chem. Soc..

[16] Yu S (2015). A promising cathode material of sodium iron-nickel hexacyanoferrate for sodium ion batteries. J. Power Sources.

[17] You Y, Wu X-L, Yin Y-X, Guo Y-G (2014). High-quality prussian blue crystals as superior cathode materials for room-temperature sodium-ion batteries. Energy Environ. Sci..

[18] Xiao P, Song J, Wang L, Goodenough J, Henkelman G (2015). Theoretical study of the structural evolution of a Na_*x*_FeMn(CN)_6_ cathode upon Na intercalation. Chem. Mater..

[19] Takachi M, Matsuda T, Moritomo Y (2013). Cobalt hexacyanoferrate as cathode material for Na^+^ secondary battery. Appl. Phys. Express.

[20] Takachi M, Matsuda T, Moritomo Y (2013). Redox reactions in prussian blue analogues against Li concentration. Jpn. J. Appl. Phys..

[21] Gotoh A (2007). Simple synthesis of three primary color nanoparticle inks of Prussian blue and its analogues. Nanotechnology.

[22] Hara S (2007). Electrochromic thin film of Prussian blue nanoparticles fabricated using wet process. Jpn. J. Appl. Phys..

[23] Magnússon RL, Kobayashi W, Takachi M, Moritomo Y (2017). Temperature effect on redox voltage in Li_*x*_Co[Fe(CN)_6_]_*y*_. AIP Adv..

[24] Izumi F, Momma K (2007). Three-dimensional visualization in powder diffraction. J. Solid State Phenom..

[25] Ravel B, Newwille M (2005). ATHENA, ARTEMIS, HEPHAESTUS: data analysis for X-ray absorption spectroscopy using IFEFFIT. J. Synchrotron. Rad..

[26] Kelly SD (2009). Comparison of EXAFS foil spectra from around the world. J. Phys. Conf. Ser..

