# Supplementary material for: Invariant nature of substituted element in metal-hexacyanoferrate
Source: Sci Rep. 2017 Oct 16;7:13225. doi: 10.1038/s41598-017-13719-z (PMC5643321; doi:10.1038/s41598-017-13719-z)
Supplement: Supplementary file 1 — Supporting information [file 41598_2017_13719_MOESM1_ESM.doc]

Supporting information

**Invariant nature of substituted element in metal-hexacyanoferrate**

**Hideharu Niwa^1*^, Wataru Kobayashi^1,2^, Takayuki Shibata^3^, Hiroaki Nitani^4^, and Yutaka Moritomo^1,2*^**

^1^Faculty of Pure and Applied Science, University of Tsukuba, Tsukuba 305-8571, Japan

^2^Tsukuba Research Center for Interdisciplinary Materials Sciences (TIMS), University of Tsukuba, Tsukuba 305-8571, Japan

^3^National Institute of Technology, Gunma College, Maebashi, Gunma 371-8530, Japan

^4^Institute of Materials Science, High Energy Accelerator Research Organization (KEK), Tsukuba 305-0801, Japan

Contact information:

Yutaka Moritomo / Hideharu Niwa

Faculty of Pure and Applied Science,

Univ. of Tsukuba, Tennodai 1-1-1, Tsukuba 305-8571, Japan

Tel +81-29-853-4337 / +81-29-853-4216

e-mail: moritomo.yutaka.gf@u.tsukuba.ac.jp / niwa.hideharu.ga@u.tsukuba.ac.jp

Fig. S1: XRD patterns of pure (*M*-HCF) and mixed (*M*_h_*M*_g_-HCF) compounds. In Mn-, MnCo-, MnNi-, Co-, CoMn-, and CoNi-HCFs, doublet features at 10.5 degree indicate trigonal structure.

Fig. S2: (a) FT[*χ*(*k*)*k*^3^]−*R* plot around the Co K-edge of CoMn-HCF. (b) FT[*χ*(*k*)*k*^3^]−*R* plot around the Mn K-edge of CoMn-HCF. Red curve is results of the least-squares fitting with the EXFAS equation in the *R* range from 1Å to 3.2Å.

Fig. S3: XANES spectra around the Fe K-edge in (a) pure (*M*-HCF) and (b) mixed (*M*_h_*M*_g_-HCF) compounds. Thick curves represent the spectra of pure compounds. Thin solid and broken curves correspond to the spectra around *M*_h_ and *M*_g_ in the mixed compounds, respectively. (c) and (d) Magnified spectra in the pre-edge region.

Table S1: The EXAFS structural parameters of pure (*M*-HCF) and mixed (*M*_h_*M*_g_-HCF) compounds. Superscript, pure, means pure compound. Superscripts, h and g, mean host and guest elements, respectively. *d*, σ^2^, *N*_N_, *E*_0_ are the interatomic distance, Debye–Waller factor for the respective path, coordination number of N, and absorption edge energy, respectively.

Table S2: The EXAFS structural parameters of pure (*M*-HCF) and mixed (*M*_h_*M*_g_-HCF) compounds. *d*, σ^2^, *E*_0_ are the interatomic distance, Debye–Waller factor for the respective path, and absorption edge energy, respectively.
